# Dynamic evolution of hepatitis C virus resistance-associated substitutions in the absence of antiviral treatment

**DOI:** 10.1038/srep41719

**Published:** 2017-01-31

**Authors:** Auda A. Eltahla, Preston Leung, Mehdi R. Pirozyan, Chaturaka Rodrigo, Jason Grebely, Tanya Applegate, Lisa Maher, Fabio Luciani, Andrew R. Lloyd, Rowena A. Bull

**Affiliations:** 1 School of Medical Sciences, Faculty of Medicine, UNSW Australia, Sydney, NSW 2052, Australia; 2 The Kirby Institute, UNSW Australia, Sydney, NSW 2052, Australia

## Abstract

Resistance against new hepatitis C virus (HCV) antivirals is an area of increasing interest. Resistance-associated substitutions (RASs) have been identified in treatment-naïve individuals, but pressures driving treatment-independent RAS emergence are poorly understood. We analysed the longitudinal evolution of RASs in twelve participants with early acute HCV infections. Full-genome deep sequences were analysed for changes in RAS frequency within NS3, NS5A and NS5B-coding regions over the course of the infection. Emergence of RASs relevant only to the polymerase non-nucleoside inhibitors (NNI) was detected, and these lay within CD8+ T-cell epitopes. Conversely, the loss of NNI RASs over time appeared likely to be driven by viral fitness constraints. These results highlight the importance of monitoring CD8+ T cell epitope-associated RASs in populations with dominant HLA types.

Antiviral therapy for hepatitis C virus (HCV) has undergone a recent revolution with the approval of several direct-acting antivirals (DAA) targeting the HCV protease (NS3), phosphoprotein (NS5A) and polymerase (NS5B). Interferon-free regimens, which contain multiple DAAs, have been approved in several countries to treat infections with different HCV genotypes^1^. Each agent has been shown to promote emergence of resistance-associated substitutions (RASs) when studied *in vitro,* and depending on the DAA, the presence of these substitutions could reduce the efficacy of antiviral treatment *in vivo*^2^. Monitoring the emergence of HCV RASs *in vivo* is therefore of particular importance to both clinical practice and public health strategies.

As a consequence of an error-prone viral polymerase, and a high replication rate, a swarm of mutations constantly get generated during HCV infection. Some of these natural mutations encode for RASs and have been reported in many studies in treatment-naïve patients^3^,^4^,^5^,^6^. Such naturally occurring RASs could negatively impact treatment success rates, particularly in patients with cirrhosis, those infected with genotype 3, and those who have failed interferon-based therapies. In the context of antiviral treatment, the emergence of RASs is initially limited by the genetic barrier to resistance, defined by the number and type of nucleotide changes required for amino acid substitutions^7^. The potential for particular RASs to persist in the host ultimately depends on the trade-off between the loss of replicative fitness and the survival advantage under strong antiviral drug selection pressure. In the absence of treatment, RASs may be retained if they increase viral fitness, are unable to revert back to wild-type^2^, or potentially if the relevant sites are under other selective pressures including host immune responses. For instance, some RAS sites within NS3 and NS5B have been shown to fall within experimentally-confirmed or predicted CD8+ T cell epitopes^6^,^8^,^9^. In addition, the prevalence of an NS5A RAS was recently shown to be associated with the host interferon λ4 (IFNL4) genotype^10^. These findings therefore suggest that both innate and adaptive immune responses play a role in the emergence of HCV DAA resistance in the absence of antiviral treatment.

As primary HCV infection is typically asymptomatic, it has been challenging to characterise the evolution of HCV mutations, and hence RASs, in the early phase of infection. Furthermore, the lack of well-characterised samples collected over the course of infection has limited longitudinal analyses of the interplay between the host immune response and viral fitness in relation to RAS development. Characterisation of the emergence of RASs in the absence of antiviral pressures is critical to our understanding of their stability within the host, and their potential influence on treatment options. The aim of this study was to examine the longitudinal emergence of RASs in the early phase of primary HCV infection. Specifically, the aim was to understand the interplay between viral replicative fitness and the host T cell responses in driving the emergence of RASs in the absence of antiviral treatment.

## Methods

### Subjects and samples

Viremic blood samples were obtained from prospective cohorts of high-risk, HCV-uninfected subjects recruited between 2005 and 2014 in NSW Australia. Sample were obtained from two cohorts; the Hepatitis C Incidence and Transmission Study in prisons (HITS-p) and in the community (HITS-c)^11^,^12^. Participants were tested every three to six months for HCV seroconversion, and then followed regularly post-infection until spontaneous clearance or persistence was determined when antiviral treatment was offered if they remained infected. For this study, twelve participants with early acute HCV infection were included. An early infection case was defined by the availability of at least one viremic sample prior to seroconversion. The estimated date of infection was calculated for each subject by subtracting the recognized mean pre-seroconversion window period of 51 days from the midpoint between the last HCV RNA-positive/HCV Ab-negative time point and the first seropositive time point^11^. Ethical approvals were obtained from the Human Research Ethics Committees of Justice Health (reference number G304/11), the New South Wales Department of Corrective Services (reference number 05/0884), and the University of New South Wales (reference numbers 05094, 08081, 13237, 09075, 14170). Written informed consent was obtained from the participants. All methods were also performed in accordance with the relevant guidelines and regulations.

### Viral genome sequencing and analysis

Viral RNA was extracted from plasma samples and amplicons covering the full HCV genome were generated either as single near-full length products, or as three overlapping fragments, as described previously^13^,^14^. Next-generation sequencing of the amplicons was conducted using either the Roche 454 FLX, or the Illumina MiSeq sequencing platform, as previously reported^11^,^13^. Sequence alignments were generated using Bowtie 2^15^ against the corresponding genotype reference sequence: GT1a (GenBank accession numbers AF009606), GT1b (AJ238799), GT2b (AB030907) and GT3a (D17763). Single-nucleotide polymorphism (SNP) analysis was performed using the Geneious software package version 8^16^ with minimum variant frequency threshold of 0.001, maximum variant P-value of 10^−6^, and a minimum coverage of 1,000 (Illumina) or 400 (Roche 454). Sixty eight positions across the genome where RASs have previously been reported^2^ were analysed. These covered RASs impacting NS3, NS5A and NS5B inhibitors. Consensus sequences for each HCV genotype were obtained from the Los Alamos Hepatitis C Sequence Database (http://hcv.lanl.gov).

### Analysis of autologous T cell responses across known RAS sites

HCV residue positions where RASs were gained over the course of infection were examined to determine whether their site of occurrence fell within potential CD8+ T cell epitopes. Potential HLA-I restricted epitopes were identified from previous experimental validation, and via bioinformatic predictions using NetMHC (www.iedb.org net). Predictions were obtained from HLA-I typing of the subject as well as from the autologous viral consensus sequence, generated via next generation sequencing. As predicted epitopes could represent false positives, strict selection criteria were applied as previously described^13^. Selected autologous HLA-1 restricted peptides were synthesised and epitope-specific interferon gamma (IFN-γ) production assessed via enzyme-linked immunospot (ELISpot) assays as previously described^11^. A positive response towards an autologous peptide was defined as >20 spot-forming units (SFU)/million peripheral blood mononuclear cells (PBMC).

## Results and Discussion

**Figure 1:**
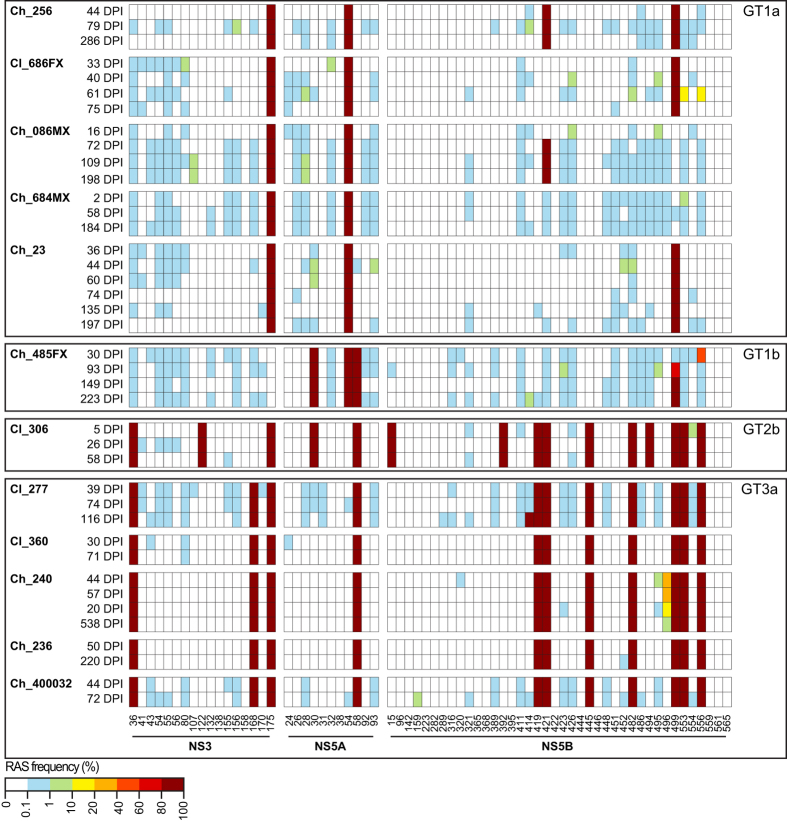
Longitudinal analysis of HCV resistance-associated substitutions (RAS) in early HCV infections. RASs are shown in longitudinally sampled infections across HCV NS3, NS5A and NS5B. Rows represent individual samples and columns indicate amino acid positions, grouped by different coding regions and numbered at the bottom. Sample identification numbers are shown together with the estimated days post infection (DPI) to the left of each row. Samples are grouped by HCV genotype (GT). The frequency of RASs is indicated by the color according to the scale bar. RASs that are fixed within an HCV genotype^34^ (i.e. consensus RASs) are presented with 100% frequency across time-points.

The longitudinal prevalence of RASs was analysed in 12 participants, each with 2–6 time-points (Fig. 1). The median number of days post-infection (DPI) at the earliest time-points was estimated at 34 (range 2–50). Four of these participants demonstrated spontaneous clearance (Cl), and eight developed chronic (Ch) infection (Fig. 1). HCV genotype distribution was GT1a (n = 5), GT1b (n = 1), GT2b (n = 1) and GT3a (n = 5).

### Longitudinal analysis of HCV RASs

Sixty-eight previously known RAS sites within NS3, NS5A and NS5B were screened in a total of 40 samples sequenced by next generation sequencing. Across the entire dataset, RASs were detected in 30/68 genome positions at frequencies >1% spanning NS3, NS5A and NS5B-coding regions (Fig. 1). While low-frequency variants (<1%) were detected within 51/68 (75%) of the examined sites at the earliest time-points, longitudinal analysis revealed that RASs within the majority of these sites (49/51, 96%) did not reach frequencies >10% over the course of the infection (Fig. 1).

Within NS3 and NS5A, all RASs detected at the consensus-level in the earliest time-points were conserved over time, and no new RASs emerged at high frequency or were lost over the course of the infection (Fig. 1). By contrast, within NS5B non-synonymous mutations resulting in emergence of consensus-level RASs M414T, A421V and V499A were observed in three participants infected with HCV GT3a, GT1a and GT1b, respectively (Fig. 1 and Table 1). Additionally, loss of consensus NS5B RASs S556G and P496S was observed in two participants infected with HCV GT1b and GT3a, respectively (Fig. 1 and Table 1). Interestingly, two NS5B RASs, A553V and S556G, were transiently detected at 61 DPI with frequencies 17–18% in a participant infected with GT1a (Cl_686FX), but these RASs were both lost within two weeks (Fig. 1). In summary, RASs affecting HCV DAAs targeting NS3 and NS5A are stable over the course of primary infection in this study, but emergence and loss of RASs were detected within NS5B of multiple genotypes.

### Factors associated with the emergence of HCV RASs

In order to examine selective pressures that could be associated with the gain of NS5B RASs, we assessed whether these substitutions fell within known, or predicted, CD8+ T cell epitopes. For the three subjects with evidence of RAS emergence and reaching fixation over time, autologous CD8+ T cell responses were tested via IFN-γ ELISpot assays. In parallel, selection by replication fitness was defined as the emergence of a mutation that was identical to the consensus genome for that sample’s corresponding genotype, which was termed to be a reversion event.

In all three cases where fixation events resulted in the gain of RASs, M414T (Cl_277), A421V (Ch_86MX) and V499A (Ch_485FX), the mutations were not reversion events, suggesting that viral fitness was not driving selection of these residues (Table 1). In relation to T cell driven pressures, M414T was identified to lie within the published HLA-A2 restricted epitope _2838_WLGNIIMYA_2846_ (RAS site underlined)^17^. When this peptide was examined by ELISpot, a moderate IFN-γ response was detected for participant Cl_277 at 74 DPI, with 20 SFU/million PBMC. At 116 DPI, the time-point at which the RAS was detected, a slightly stronger T cell response was detected, with 45 SFU/million PBMC. These findings support the hypothesis that CD8+ T cell responses exerted selective pressure on this RAS site (Table 1).

The second RAS, A421V, reached fixation in participant Ch_086MX at 72 DPI, and is recognised to lie within an HLA-B27-restricted CD8+ T cell epitope _2841_ARMILMTHF_2849_^18^,^19^. In the subject analysed here (Ch_086MX), the autologous epitope present in the transmitted/founder virus that established the infection differed from this known epitope at two positions, 2844 and 2845 (_2841_ARMVMMTHF_2849_). Experimental validation of this epitope was positive at 72 DPI, with a low CD8+ T cell frequency response of 25 SFU/million PBMCs (Table 1). When the putative escape variant epitope, _2841_VRMVMMTHF_2849_ was tested using ELISpot, no response could be detected.

Despite the lack of longitudinal evidence of an ongoing T cell response against this epitope, this immunodominant epitope is reported to be targeted by almost all subjects with the HLA-B27 genotype that are infected with HCV, and the A421V substitution has been shown to be HLA-driven^18^,^19^. These data support the hypothesis that the A421V substitution in subject Ch_086MX could be driven by T cell responses.

To further validate the hypothesis that the evolution of RASs within a single infection could be driven by the T cell response, two participants, infected with the same transmitted founder virus and with longitudinal information on viral mutations were examined. Participants Ch_086MX and Ch_684MX were siblings who concurrently became infected. The consensus viral sequences for these subjects shared 99.9% nucleotide similarity across the transmitted founder virus genome (Fig. 1). At the earliest time-points analysed (16 DPI and 2 DPI for Ch_086MX and Ch_684MX, respectively), both subjects carried the variant sequence _2841_ARMVMMTHF_2849_ (Fig. 1), which is the known HLA-B27-restricted CD8+ T cell epitope. Notably, only the virus in the the subject carrying the HLA-B27:05 allele developed the A421V substitution, while the sibling carried the HLA-B27:10, for which the predicted binding score for the same epitope is significantly lower (157 μM and 2,619 μM, respectively). Despite this, the _2841_ARMVMMTHF_2849_ peptide was tested via ELISpot in Ch_684MX and found to be weakly positive (30 SFU/million PBMC). A number of factors could explain the lack of emergence of A421V in subject Ch_684MX, including differences in peptide affinity or T cell exhaustion^20^. Investigation of these possibilities was beyond the scope of this study.

The RAS V499A was detected in participant Ch_485FX, a site which falls within the known HLA-B57-restricted epitope, _2912_LGVPPLRAWR_2921_^21^. However, the autologous circulating virus carried a substitution at position 2919, and the predicted epitope was the nonamer _2913_GVPPLRVWR_2921_. This subject was not HLA-B57 positive, and the only HLA allele of Ch_485FX predicted to bind to this peptide was HLA-A01:01, which had an IC_50_ score of 22 mM. The ELISpot response against the predicted transmitted founder derived, HLA-A01:01-restricted autologous epitope, _2913_GVPPLRVWR_2921_ was negative (Table 1). Therefore, the role of T cell responses in driving the emergence of this RAS could not be ascertained.

Overall, these results suggest that RASs M414T and A421V emerged in the early phase of HCV infection as a result of T cell responses in participants carrying relevant HLA-I alleles.

### Factors associated with the loss of HCV RASs

Unlike emerging RASs, all mutations which resulted in the loss of HCV RASs in this study were reversion events (Table 1). The loss of RAS S556G was observed as a result of a fixation event around 93 DPI in the same GT1b infected participant in whom another RAS, V499A, emerged (Ch_485FX, Fig. 1). Peptides encompassing this RAS were screened for CD8 T cell responses in previous studies, and found to be negative^22^,^23^. The loss of the second RAS, P496S, was observed in a GT3a infected participant (Ch_240) as a gradual decrease in frequency from 33% to 1.5% between 44 DPI and 538 DPI (Fig. 1). The site of this RAS, P496, was found to be conserved across all HCV GTs. This suggests that the loss of the RAS could be driven by fitness costs of the substitution. In the participant where two RASs emerged at low frequency, Cl_686FX, both RASs also reverted to the consensus sequence (Table 1). One of these RASs, S556G, overlapped with that detected in another participant, Ch_485FX (Fig. 1). The second RAS, A553V, fell within a predicted HLA-A01:01 restricted epitope, however, no response could be detected against this epitope in the ELISpot assays (Table 1). Overall, these results indicate that T cell responses do not appear to be driving the loss of HCV RASs detected in early acute HCV infection. Instead, reversion of residues carried by the virus towards consensus sequences indicates that the observed loss of RASs could be driven by reduced viral fitness.

The occurrence of RASs in the transmitted founder sequences within these subjects could be attributed to immune responses within the original host (i.e the transmission source) that were not investigated in this study, or to bottleneck selection in the new host (i.e the recipient) during viral transmission. They could also be due to transmission of drug-resistance variants from a treatment-experienced source. However, the participants in this study were recruited and sampled prior to the availability of HCV NNIs, and while the participants were not part of DAA clinical trials, these were being conducted concurrently in the Australian community, and there remains the possibility that the source was treatment-experienced.

### Relevance of detected evolving RASs

All six RASs with longitudinal evolution in this study are relevant to one class of DAAs; non-nucleoside inhibitors (NNI) of the HCV NS5B. The RAS V499A, causes a minor reduction to some thumb-binding HCV NNIs^24^ but both none of those in development. A421V is associated with resistance to thumb-binding NS5B inhibitors, such as beclabuvir^25^; A421V, was identified upon treatment of GT1 patients with beclabuvir^25^, and with the combination daclatasvir/asunaprevir/beclabuvir, but had no significant association with virologic outcome^26^. M414T confers resistance to palm-binders, including the recently approved NNI dasabuvir^27^. Viral breakthrough and relapse after treatment in an IFN-free combination including dasabuvir have been associated with substitutions with M414I/T^28^,^29^,^30^. It should be noted, however, that the M414T substitution is observed in a GT3a infected participant and dasabuvir is known to have significantly reduced activity against GT3a in general^27^.

The fitness of these RASs in cell culture models is variable, and depends on the examined genotypes. A421V does not seem to impact the replication of GT1a^31^ or GT1b^32^ replicons, whilst M414T affects the fitness of GT1b but not GT1a in replicon studies^27^. V499A was shown to impact the replication of GT1b *in vitro*^24^. This RAS, however, is not uncommon in GT1b infections, and therefore its impact on viral fitness could not be ascertained.

Of the RASs that were observed to be lost over the analysed course of infection, A553T (in GT1a), A553V (in GT1b) and S556G (in both GT1a and GT1b) are key substitutions associated with reduced response to dasabuvir *in vitro* and *in vivo*^27^. These substitutions have been associated with reduced fitness of GT1 HCV *in vitro*^27^, potentially explaining their loss over time.

### Summary

Selection of HCV RASs via immune factors has been proposed in a number of studies. Emergence and loss of an NS3 RAS, R155K, has been previously reported in a single patient co-infected with both HIV-1 and GT1a HCV when sampled over time^33^. This site was later confirmed to fall within a CD8+ T cell epitope and the RAS was proposed to be associated with loss of recognition by these cells^9^. Resistance to other HCV DAAs has been proposed to arise through immune-driven selection in some putative HLA-restricted epitopes^8^, but these responses were not verified experimentally. Our study describes the evolution of six RASs during early acute HCV infections with three different genotypes, and proposes that immune selection pressures contribute to the emergence of HCV RASs in early HCV infections, particularly within NS5B, even in the absence of treatment. This study also supports previous reports highlighting the incurred fitness cost of RASs, and suggests that this cost contributes to their loss. These results suggest that populations with particular HLA dominant types should be closely monitored for changes in prevalence of immune-related RASs.

## Additional Information

**How to cite this article:** Eltahla, A. A. *et al*. Dynamic evolution of hepatitis C virus resistance-associated substitutions in the absence of antiviral treatment. *Sci. Rep.* **7**, 41719; doi: 10.1038/srep41719 (2017).

**Publisher's note:** Springer Nature remains neutral with regard to jurisdictional claims in published maps and institutional affiliations.

## Figures and Tables

**Table 1:** Summary of the RAS analysis in this study with corresponding ELISpot responses.

Participant	HCV Genotype	Substitution detected	RAS outcome^a^	Position^b^	Reversion	CD8 T cell epitope analysis			
						Epitope sequence	HLA	Result (SFU/10^6^ PBMC)^c^	Time-point (DPI)^d^
Ch_086MX	1a	A421V	Gain	2841	No	_2841_ARMVMMTHF_2849_	HLA-B27:05	25	72
Ch_485FX	1b	V499A	Gain	2918	No	_2913_GVPPLRVWR_2921_	HLA-A01:01	Negative	79
		S556G	Loss	2975	Yes	—	—	—	—
Cl_277	3a	M414T	Gain	2844	No	_2838_WLGNIIMYA_2846_	HLA-A02:01	45	116
Ch_240	3a	P496S	Loss	2926	Yes	—	—	—	—
Cl_686FX	1a	A553V	Gain/Loss	2973	Yes	_2967_LSGWFTAGY_2975_	HLA-A01:01	Negative	117
		S556G	Gain/Loss	2976	Yes	—	—	—	—

^a^RAS; resistance-associated substitution. Gain or loss or RAS is determined longitudinally with reference to the earliest sample.

^b^Amino acid position with reference to strain H77 (AF009606) for GT1a, Con1 (AJ238799) for GT1b, and NZL1 (D17763) for GT3a.

^c^Spot-forming units per million peripheral blood mononuclear cells.

^d^Estimated days post infection (DPI) at which analysis was performed.
